# Surgical gestures as a method to quantify surgical performance and predict patient outcomes

**DOI:** 10.1038/s41746-022-00738-y

**Published:** 2022-12-22

**Authors:** Runzhuo Ma, Ashwin Ramaswamy, Jiashu Xu, Loc Trinh, Dani Kiyasseh, Timothy N. Chu, Elyssa Y. Wong, Ryan S. Lee, Ivan Rodriguez, Gina DeMeo, Aditya Desai, Maxwell X. Otiato, Sidney I. Roberts, Jessica H. Nguyen, Jasper Laca, Yan Liu, Katarina Urbanova, Christian Wagner, Animashree Anandkumar, Jim C. Hu, Andrew J. Hung

**Affiliations:** 1 Center for Robotic Simulation & Education, Catherine & Joseph Aresty Department of Urology, USC Institute of Urology, University of Southern California, Los Angeles, CA USA; 2 Department of Urology, Weill Cornell Medicine, New York, NY USA; 3 Computer Science Department, Viterbi School of Engineering, University of Southern California, Los Angeles, CA USA; 4 Department of Computing & Mathematical Sciences, California Institute of Technology, Pasadena, CA USA; 5 Department of Urology and Urologic Oncology, St. Antonius-Hospital, Gronau, Germany

**Keywords:** Outcomes research, Health services

## Abstract

How well a surgery is performed impacts a patient’s outcomes; however, objective quantification of performance remains an unsolved challenge. Deconstructing a procedure into discrete instrument-tissue “gestures” is a emerging way to understand surgery. To establish this paradigm in a procedure where performance is the most important factor for patient outcomes, we identify 34,323 individual gestures performed in 80 nerve-sparing robot-assisted radical prostatectomies from two international medical centers. Gestures are classified into nine distinct dissection gestures (e.g., hot cut) and four supporting gestures (e.g., retraction). Our primary outcome is to identify factors impacting a patient’s 1-year erectile function (EF) recovery after radical prostatectomy. We find that less use of hot cut and more use of peel/push are statistically associated with better chance of 1-year EF recovery. Our results also show interactions between surgeon experience and gesture types—similar gesture selection resulted in different EF recovery rates dependent on surgeon experience. To further validate this framework, two teams independently constructe distinct machine learning models using gesture sequences vs. traditional clinical features to predict 1-year EF. In both models, gesture sequences are able to better predict 1-year EF (Team 1: AUC 0.77, 95% CI 0.73–0.81; Team 2: AUC 0.68, 95% CI 0.66–0.70) than traditional clinical features (Team 1: AUC 0.69, 95% CI 0.65–0.73; Team 2: AUC 0.65, 95% CI 0.62–0.68). Our results suggest that gestures provide a granular method to objectively indicate surgical performance and outcomes. Application of this methodology to other surgeries may lead to discoveries on methods to improve surgery.

## Introduction

In the past decade, mounting evidence has demonstrated that surgical performance significantly impacts surgical outcomes^1,2^. For example, lower operative skill in laparoscopic gastric bypass is associated with higher complication rates, higher mortality rates, longer operations, and higher rates of reoperation and readmission^1^. To enhance surgical outcomes, one must first quantify surgical performance^3^. However, it still remains challenging to objectively achieve so.

**Fig. 1:**
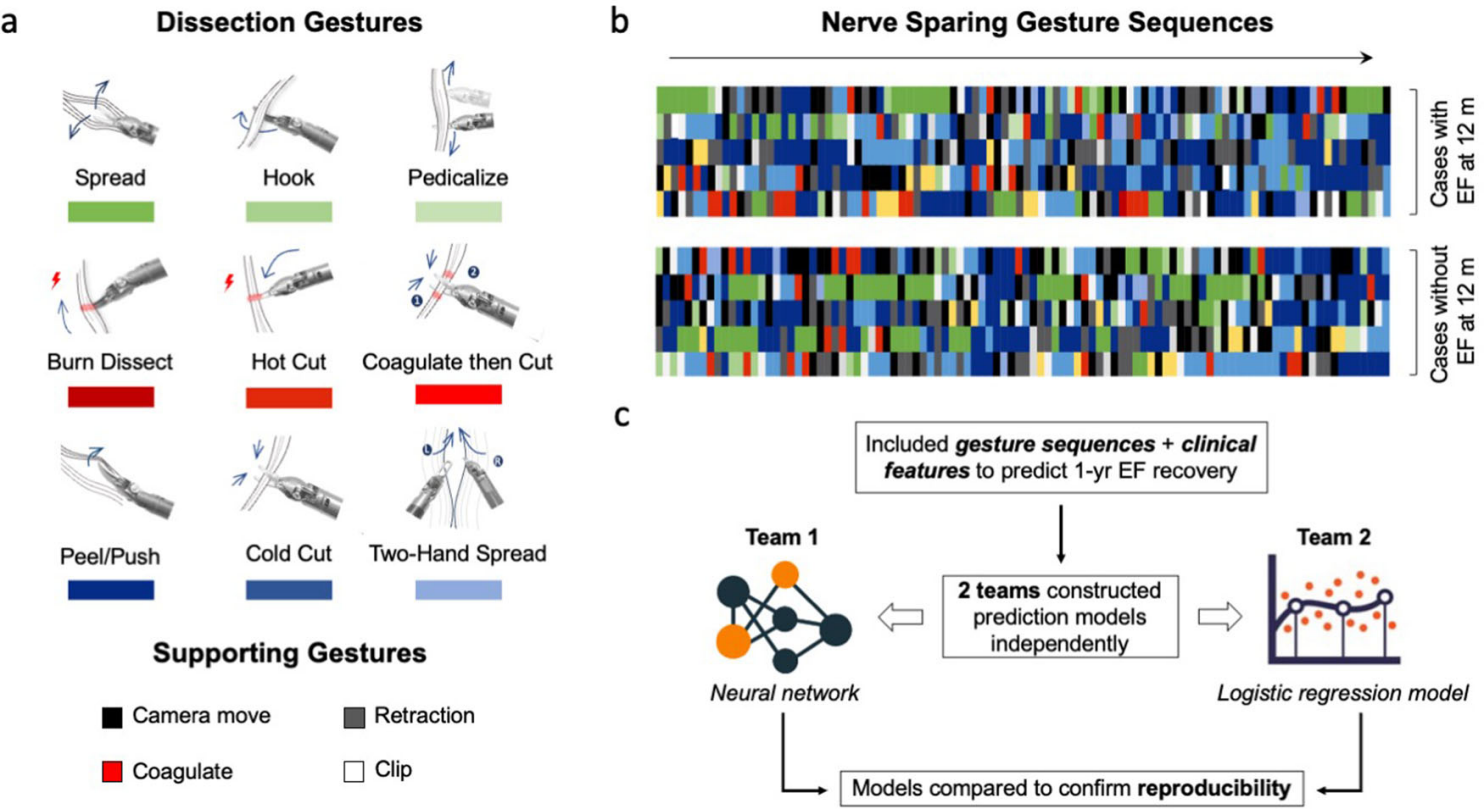
Dissection gestures and the study design. **a** Gesture classification, including 9 dissection gestures and 4 supporting gestures. **b** Color-coded nerve-sparing gesture sequences (showing only the first 100 gestures). Colors represented corresponding gestures in **a**. **c** One-year EF recovery prediction model construction process. Two teams independently constructed and tested their prediction models to confirm the reproducibility of results.

Surgical gestures, defined as the smallest meaningful interaction of a surgical instrument with human tissue^4,5^, are a novel approach to deconstruct surgery. They have the potential to objectively quantify surgery meanwhile provide actionable feedback for trainees. Previously, we developed a dissection surgical gesture classification system consisting of nine distinct dissection gestures (e.g., cold cut) and four supporting gestures (e.g., retraction) (Fig. 1a)^5^. We found that different selections of surgical gestures during the hilar dissection step of robot-assisted partial nephrectomy can distinguish the expertise of surgeons^5^. However, it is still unclear whether different surgical gestures are associated with patient outcomes after surgery.

Robot-assisted radical prostatectomy (RARP), the most common treatment for prostate cancer, is an ideal test case to evaluate whether surgical gestures relate to a patient’s outcomes because it has a concrete, easily measured functional outcome that is highly associated with surgical performance^6^. Erectile dysfunction after RARP has a profound impact on a man’s quality of life and over 60% of men experience this long-term complication due to injury of the nerves that run alongside the prostate^7^. During nerve spare (NS), surgeons gently peel these nerves off from the prostate. Minute changes in a surgeon’s dissection technique can have a major impact on a patient’s potency recovery^8^. Ample evidence suggests that a surgeon’s performance matters: while patients of the top-tier surgeons have a nearly 50% chance of recovering potency, patients of the bottom-tier surgeons have less than a 20% chance^9^.

Given the association between the quality of nerve sparing and risk of postoperative erectile dysfunction, we primarily aim to examine whether the gestures used during the NS step of RARP can predict rates of ED after surgery. The secondary objective is to study surgical gesture selection by surgeons of varying experience levels to deepen our understanding of different dissection techniques of nerve sparing. We hypothesize that surgical gestures can be used as an effective tool to quantify technical skills and potentially indicate surgical outcomes.

In this international bi-center study, we find that less use of hot cut and more use of peel/push during NS are associated with a better chance of 1-year EF recovery. Moreover, using dissection gesture sequences during NS, ML models can be constructed to accurately predict EF recovery. In addition, we find surgeons with different experience levels use different surgical gestures during NS. These results suggest that breaking down surgery to the level of surgical gestures can serve as a novel method to measure surgical performance, which may have wider applications to different surgical specialties to predict surgical outcomes and give actionable feedback.

## Results

### Baseline cohort data

**Fig. 2:**
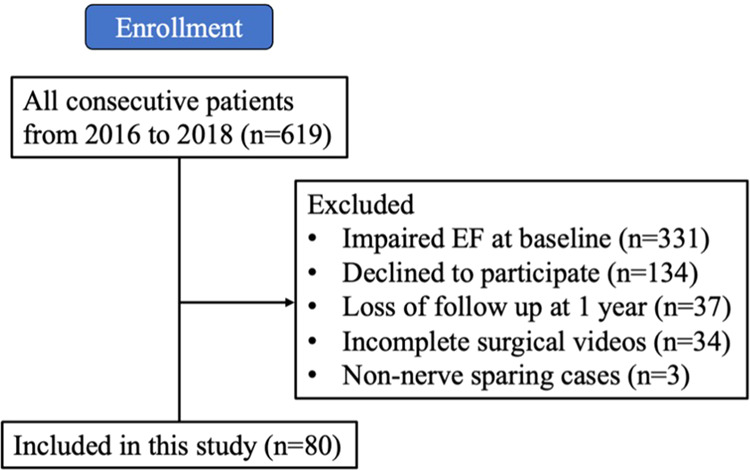
Flowchart of patient enrollment. Enrollment of 80 RARP cases.

Six hundred nineteen consecutive RARP cases were candidates for this study, and eventually 80 cases from 21 surgeons from 2 international surgical centers fulfilled our inclusion/exclusion criteria (Fig. 2). Most patients were excluded because they did not have baseline erectile function to be preserved during surgery. The median prior robotic surgical caseload of these 21 practicing surgeons was 450 (range 100–5800) cases. There was a gap in robotic surgical experience between a group of 6 super-experts (median 3000 cases, range 2000–5800) and a group of 15 experts (median 275 cases, range 100–750) (Supplementary Table 1).

Overall, 1-year postoperative EF recovery rate was 34/80 (43%). Patients who recovered EF were significantly younger (*p* = 0.02, Chi-square test) and had better American Society of Anesthesiology (ASA) physical status (*p* = 0.03, Chi-square test) (Table 1). Patients who recovered EF had a greater proportion of full nerve sparing (76.5% vs 69.6%), although this was not statistically significant (*p* = 0.49, Chi-square test).

**Table 1:** Comparison of clinical features between the EF recovery group and the no EF recovery group at 1 year after RARP.

Features	No EF recovered at 1 year Median (IQR) / Count (%) (*N* = 46)	EF recovered at 1 year Median (IQR) / Count (%) (*N* = 34)	*p*-value
Patient factors			
Age, year	65 (61–69)	61 (58–65)	0.02
BMI, kg/m^2^	27.4 (25.9–29.0)	28.7 (25.3–30.1)	0.69
Pre-op SHIM score	24 (21–25)	24 (22–25)	0.36
PSA, ng/mL	6.8 (5.4–11.2)	7.7 (5.2–9.8)	0.74
ASA			0.03
I	3 (6.5%)	8 (23.5%)	
≥II	43 (93.5%)	26 (76.5%)	
Pre-op Gleason score			0.22
6 (ISUP 1)	12 (26.1%)	8 (23.5%)	
7 (ISUP 2/3)	22 (47.8%)	22 (64.7%)	
≥8 (ISUP 4/5)	12 (26.1%)	4 (11.8%)	
Post-op Gleason score			0.19
6 (ISUP 1)	4 (8,7%)	6 (17.6%)	
7 (ISUP 2/3)	32 (69.6%)	25 (73.5%)	
≥8 (ISUP 4/5)	10 (21.7%)	3 (8.8%)	
Pathological tumor stage			0.65
pT2	22 (47.8%)	18 (52.9%)	
≥pT3	24 (52.2%)	16 (47.1%)	
Prostate volume, g	48 (34–54)	39 (32–56)	0.35
Treatment factors			
Nerve-sparing extent			0.49
Partial	14 (30.4%)	8 (23.5%)	
Full	32 (69.6%)	26 (76.5%)	

Continuous variables were compared by Mann–Whitney U test and reported as median (IQR). Categorical variables were compared by Chi-square test or Fisher exact test as indicated.

*ASA* American Society of Anesthesiology physical status classification system, *BMI* body mass index, *IQR* interquartile range, *SHIM* Sexual Health Inventory for Men, *ISUP* International Society of Urological Pathology, *PSA* prostate specific antigen.

### Identify surgical gestures utilized in NS

A median of 438 discrete gestures (IQR 254–559) was identified per NS case. Active dissection gestures consisted 65.7% of all gestures, and supporting gestures consisted of the other 34.3% (Table 2).

**Table 2:** Breakdown of different gestures used in the nerve-sparing step.

Gesture	Number	Proportion
Dissection	22,566	65.7%
Peel/push	11,881	34.6%
Cold cut	6823	19.9%
Spread	1711	5.0%
Hook	963	2.8%
Hot cut	599	1.7%
Pedicalize	211	0.6%
Two-hand spread	207	0.6%
Burn	105	0.3%
Coagulation then cut	66	0.2%
Supporting	11,757	34.3%
Camera move	5204	15.2%
Retraction	4639	13.5%
Coagulation	1196	3.5%
Clip	718	2.1%

### Dissection gesture sequences and 1-year EF recovery

**Fig. 3:**
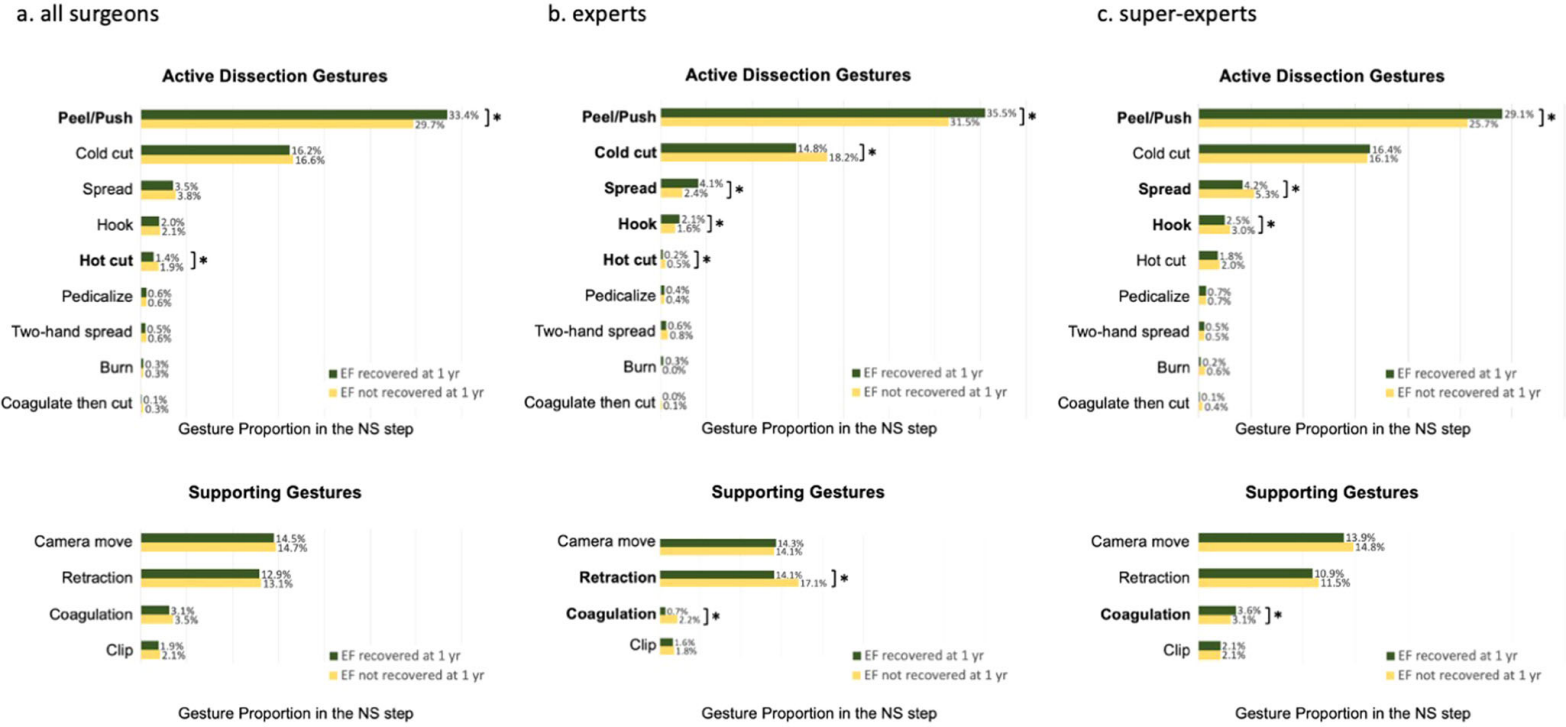
Comparison of surgical gestures in the nerve-sparing step between patients who recovered erectile function (EF) at 1 year and patients who did not recover EF at 1 year (*p < 0.05, generalized linear mixed model). **a** The whole cohort; **b** expert group; **c** super-expert group.

To assess whether a gesture type was significantly related to 1-year EF recovery, the proportion of each type of gesture within a case between EF-recovered and non-recovered patients was compared. Patients who recovered EF had less hot cut (median 1.4% vs 1.9%, *p* = 0.016, generalized linear mixed model [GLMM]) but more peel/push (median 33.4% vs 29.7%, *p* < 0.001, GLMM) (Fig. 3a). To confirm the results, we did subgroup analyses in the expert (Fig. 3b) and super-expert group (Fig. 3c), respectively. In both groups, patients who recovered EF had more peel/push (*p* ≤ 0.001, GLMM). Hot cut usage was only significant in the expert group, where patients who recovered EF had more hot cut (*p* = 0.001, GLMM). In addition, patients who recovered 1-year EF had less cold cut, more spread, more hook, less retraction, and less coagulation in the expert group (all *p* < 0.05, GLMM). In the super-expert group, patient who recovered 1-year EF had less spread, less hook, and more coagulation (all *p* < 0.05, GLMM).

**Fig. 4:**
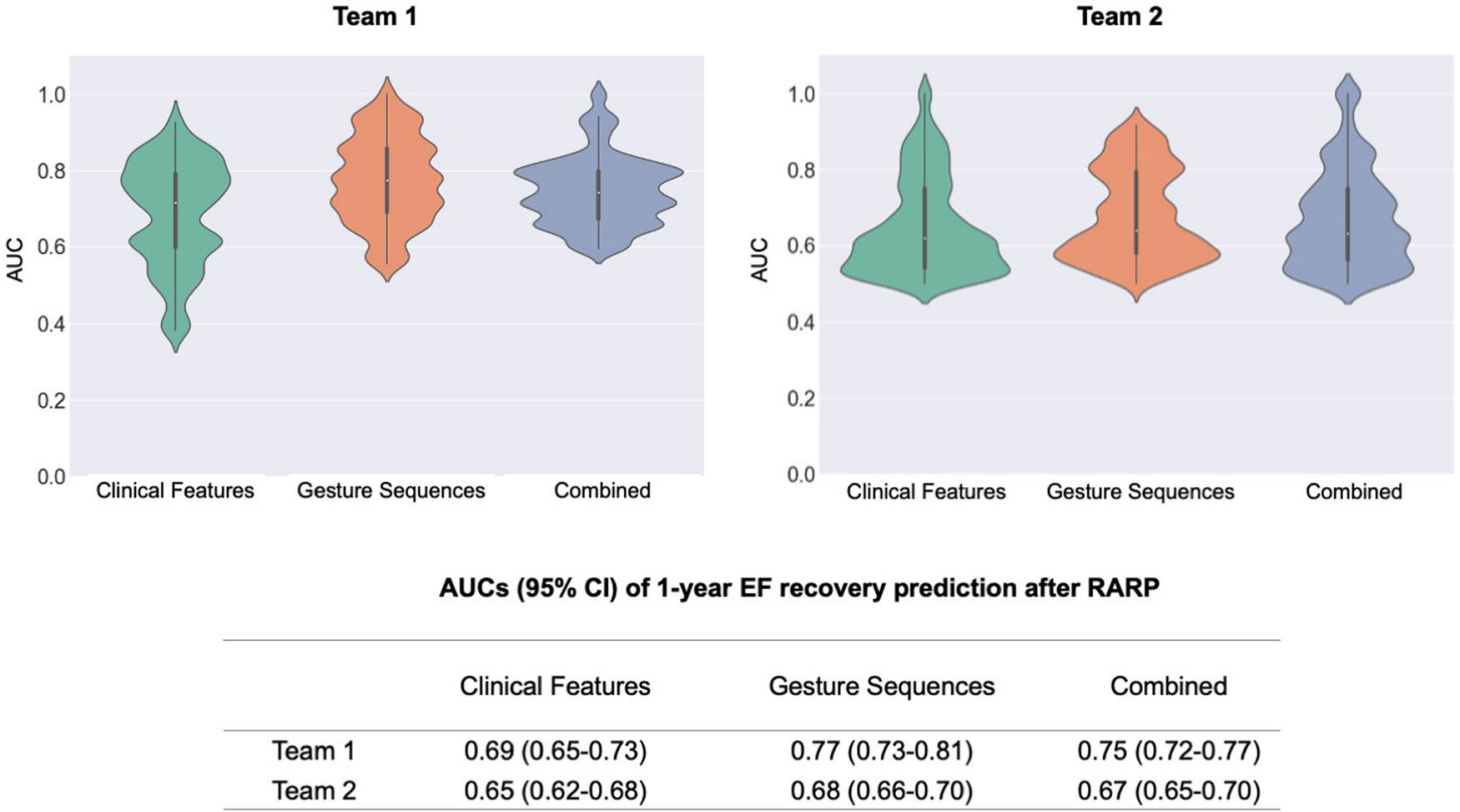
Prediction model performance. Violin plots showing the performance of 1-year EF recovery prediction models.

Gesture sequences and clinical features were then used by two teams to independently construct machine learning (ML) prediction models for 1-year EF recovery, to ensure the reproducibility of the outcomes. When including surgical gesture sequences alone, both models achieved a moderately-high ability to predict 1-year EF recovery (AUC: Team 1: 0.77, 95% CI 0.73–0.81; Team 2: 0.68, 95% CI 0.66–0.70), which surpassed clinical features alone (AUC, Team 1: 0.69, 95% CI 0.65–0.73; Team 2: 0.65, 95% CI 0.62–0.68). When models included both surgical gestures and clinical features (AUC, Team 1: 0.75, 95% CI 0.72–0.77; Team 2: 0.67, 95% CI 0.65–0.70), the models performed similarly to those that included surgical gestures alone (Fig. 4).

**Fig. 5:**
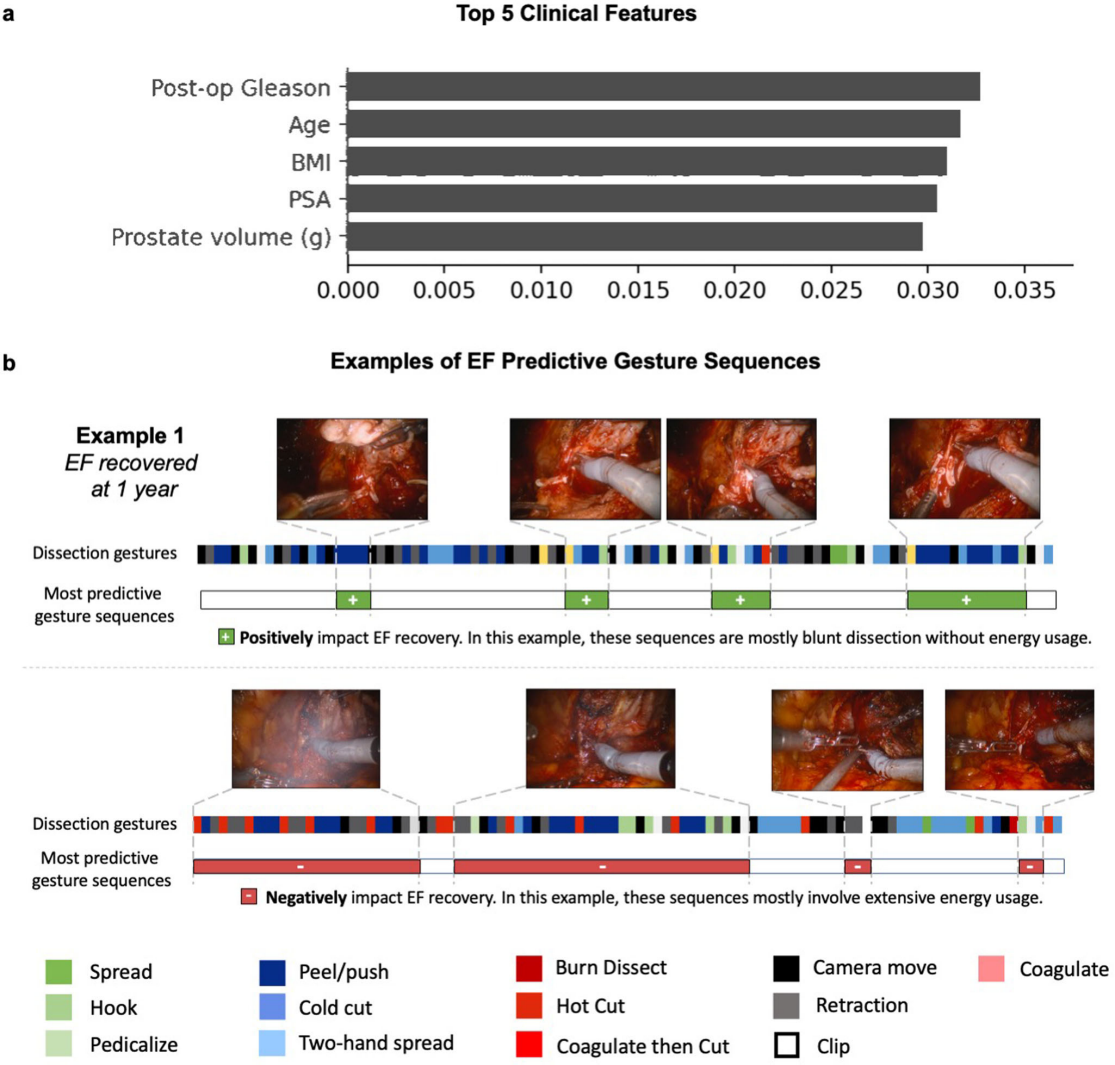
Important clinical features and gesture sequences for 1-year EF prediction. **a** Important clinical features; **b** examples of important surgical gesture sequences.

To understand how these models make predictions, we picked Team 1’s model due to its better performance and ranked the important clinical features for 1-year EF prediction (Fig. 5a), which were Gleason score, age, BMI, PSA, and prostate volume. We also outputted important gesture sequences positively or negatively associated with 1-year EF recovery (Fig. 5b).

To rule out the possibility that the ML models simply learned the prediction of 1-year EF recovery by the number of gestures used during NS rather than truly learned from the gesture sequences themselves, we ranked 80 cases based on the number of gestures and categorize cases into four quartiles. We found similar 1-year EF recovery rate across quartiles (*p* = 0.66, Chi-square test, Supplementary Table 2).

### Gesture selections between surgeons of different experience levels

Super-experts used fewer gestures than experts (median 317 vs 530, *p* = 0.014, Mann–Whitney U test) during the NS step. This trend was present for both the active dissection gestures (i.e., peel/push) and supporting gestures (i.e., camera move, retraction) (Table 3).

**Table 3:** Gesture number comparison between super-experts and experts in nerve sparing.

	Super-experts cases *N* = 53	Experts cases *N* = 27	*p*-value
Total gesture/case	317 (237–525)	530 (439–722)	0.01
Peel/push	86 (61–137)	162 (117–278)	0.03
Cold cut	64 (33–101)	100 (43–122)	0.20
Spread	13 (6–23)	18 (6–35)	0.75
Hook	9 (4–18)	13 (6–24)	0.70
Hot cut	1 (0–5)	1 (0–11)	0.17
Two-hand spread	1 (0–2)	1 (0–4)	0.37
Coagulation then cut	0 (0–0)	0 (0–0)	0.45
Pediclize	1 (0–4)	0 (0–5)	0.94
Burn	0 (0–0)	0 (0–0)	0.35
Camera move	45 (37–59)	92 (62–106)	0.02
Retraction	36 (25–52)	83 (64–110)	<0.01
Coagulation	9 (3–29)	11 (7–22)	0.85
Clip	7 (5–11)	8 (6–16)	0.38

Variables were compared by Mann–Whitney U test and reported as median (interquartile range).

When comparing gesture proportions utilized in the NS, we found that super-experts utilized more cold cut (median 18.0% vs 13.0%, *p* = 0.001, GLMM), more coagulation (median 3.5% vs 2.0%, *p* = 0.005, GLMM), but less peel/push (median 27.0% vs 34.0%, *p* = 0.024, GLMM) and less retraction adjustments (median 10.5% vs 16.0%, *p* = 0.001, GLMM).

Notably, the reported EF recovery rate was similar among patients operated by super-experts (23/53, 43.4%) compared to patients operated by experts (11/27, 40.7%, *p* = 0.82, Chi-square test). The clinical features of these two groups of patients were also similar (Supplementary Table 3).

## Discussion

In this international bi-center study, we demonstrated (a) less use of hot cut and more use of peel/push were associated with a better chance of 1-year EF recovery; (b) surgical gesture sequences can successfully predict 1-year EF recovery after RARP; and (c) surgical gesture selections were associated with surgeon experience levels. In addition, we had two teams independently confirmed the relationship between surgical gesture sequences and surgical outcomes. This dual-effort method has been rarely conducted in the clinical literature, although it has been widely advocated by the ML research community, for the purpose of increasing robustness and confirming reproducibility of ML findings^10,11^. These findings suggest that surgical gestures can serve as a novel method to quantify surgical performance and predict functional outcomes after RARP.

In this study we demonstrate an association between surgical gestures and surgical outcomes. Our results indicate that less hot cut in NS is associated with better potency recovery, especially in the expert group (rather than super-experts). This is consistent with prior studies which have reported that extensive energy use in NS has a detrimental effect on the nearby neurovascular bundles, thus impacting EF recovery^8,12^. More peel/push was associated with better potency recovery, which was confirmed in both the expert and super-expert groups. We believe peel/push is the appropriate gesture for finding the correct dissection plane during NS, which in turn can result in better outcomes. Of note, the results also showed interactions between surgeon expertise and gesture types—the same types of gestures utilized by surgeons with different experience levels can have different impact on EF recovery. For example, in the expert group, more spread, more hook, and less coagulation were associated with a higher chance of EF recovery, while in the super-expert group, less spread, less hook, and more coagulation were associated with a higher chance of EF recovery. These findings indicate that not only the types of gestures matter for outcomes, but also likely the execution and context of gestures. In a recent study we found that the efficacy and error rates of the same type of gestures were different between novices, intermediates, and experts in the dry lab setting^13^. Our next step will explore how these differences impact surgical outcomes in live surgeries.

The same concept should be applicable to other surgical procedures—by deconstructing surgery into gestures, the impact of different gestures on surgical outcomes can be studied quantifiably and objectively. Objectively assessing and quantifying surgery has conventionally been challenging. A common solution is to use objective assessment tools such as GEARS or DART to evaluate surgical skills^14–16^. Unfortunately, these tools suffer from subjectivity and do not capture surgical data at its most granular level^17^. An alternative method of quantifying surgical performance is by using automated performance metrics (APMs), such as the kinematic data of instruments^18^. APMs have been able to distinguish expertise and predict patient outcomes^19,20^. But one drawback of APMs, which are largely measures of surgeon efficiency, is that they are difficult to translate to actionable feedback^18,19,21^. Surgical gestures have the potential to objectively quantify surgery meanwhile provide actionable feedback for trainees. These metrics evaluate surgeon performance differently and contain related yet different information. Gestures comprehensively deconstruct surgical action in the context of instrument-tissue interaction based on surgical videos; kinematics provide summarized information about instrument movement based on their coordinates, which may reflect instrument efficiency more. These different assessment methods should complement each other to draw a fuller picture of surgical performance.

Incorporating surgical gestures into ML models effectively predicted postoperative 1-year EF recovery. To confirm the reproducibility of our findings, two ML teams independently constructed and evaluated two prediction models. Both teams confirmed that there were informative signals within the sequence of surgical gestures that could predict EF recovery with moderate-strong AUCs. Different surgical gesture types used in NS (e.g., the proportion of hot cut) can partly explain how the models made the predictions. In addition, ML models can also utilize the temporal (sequential) information of surgical gestures (i.e., the order of surgical gestures) which is difficult to capture by traditional statistical methods. Of note, Team 1, who used a transformer-based model, which exploited full-range temporal (sequence) information (the entire sequence), achieved higher AUCs than Team 2, who used a logistic regression, which exploited short-range temporal information (splitting the entire sequence into nonoverlapping segments of 20 gestures). This may indicate that not only the type of gestures, but also the combination and ordering of gestures together plays a role in determining patient outcomes.

Our previous study found that super-experts took a shorter time to complete NS and had better bimanual dexterity compared to experts during NS^22^. Here, using the dissection gesture classification system, we confirmed that super-experts were faster and more efficient (i.e., utilized fewer gestures). When comparing the dissection gesture proportion utilized by super-experts and experts, we found that super-experts chose different gestures compared to experts. This implies the potential use of surgical gestures to distinguish expertise.

As for clinical features, we found that EF-recovered patients were younger and had better overall conditions, which are consistent with previous publications^23,24^. Using clinical features alone to predict 1-year EF recovery achieved modest AUCs. Prior publications suggested that the baseline potency status of patients is a critical factor for EF recovery after RARP^23,24^. It is worth noting that all cases included in this study had intact preoperative EF and very high sexual health inventory for men (SHIM) scores (median 24, on a scale of 25), which may have mitigated the impact of patient factors on EF recovery prediction.

The findings in this study have important clinical implications. In the absence of an ML-based predictive system, surgeons can only receive feedback on patient outcomes such as erectile function months to years postoperatively. This temporal misalignment (between surgery to outcome) makes it difficult to assess how their actions today will impact the patient down the line. With the trained ML model presented in our paper, there is the possibility for the provision of feedback immediately after surgery—which may enable surgeons to quickly incorporate improvements into their subsequent surgeries. In addition, our group has recently constructed an ML algorithm to automate the task of gesture recognition and classification^25^. Combining with the ML model in the current study, there is the potential to fully automate the whole process—from surgical video annotation to patient outcome prediction—directly predicting patient outcomes in real-time. Our future work will be devoted to the interpretability of the model, in order to pinpoint specific dissection gesture sequences important for patient outcomes, so that more actionable feedback can be provided for training surgeons.

The present study has a few limitations. First, the sample size was relatively small, which can be expanded in the future. Nonetheless, we included data from two institutions to address generalizability. Second, we did not consider the context of the surgical gestures exerted during NS. Future studies can attribute gestures to specific anatomy (e.g., pedicles, lateral fascia, etc.) and study if the effects are similar. Third, this study only used one type of surgical procedure (i.e., NS) and the findings remain to be validated in multiple procedures across specialties. Finally, case complexity was not adjusted in the current study due to the lack of an objective measurement of case complexity. It remains to be a confounding factor for the associations between surgical gestures and surgical outcomes.

In summary, we find that dissection gestures executed during NS were predictive of EF recovery after RARP. Less use of hot cut and more use of peel/push are associated with better chance of EF recovery. ML models are constructed to accurately predict EF recovery. In addition, we correlate surgical gestures with surgeon experience. These findings implicate that deconstructing surgery to the granularity of surgical gestures can serve as a novel method to quantify surgical performance, which may potentially have a wider application to various surgical specialties to predict surgical outcomes and provide actionable feedback.

## Methods

### Study cohort and design

Under institutional review boards approval from the University of Southern California and St. Antonius-Hospital, men who underwent primary RARP from July 2016 to November 2018 from these two international institutions were prospectively collected and included in this study if the following were present: (a) an intact baseline EF; (b) complete NS surgical video footage; and (c) ≥ 1-year postoperative follow-up. Bilateral non-nerve-sparing cases were excluded. Written consents were obtained from all patients included in this study. The primary outcome was 1-year EF recovery after RARP. Intact baseline EF and 1-year EF recovery were both defined as achieving erections firm enough for sexual intercourse in >50% of attempts (score of ≥4 on the 2nd questions of the SHIM) with or without phosphodiesterase type 5 inhibitors^26^.

NS of included cases were performed by advanced surgical fellows and faculty surgeons. Surgeons were separated into two surgical experience levels based on previous publications: experts who had performed 100–1999 robotic cases and super-experts who had performed ≥2000 robotic cases^22,27^.

Clinical data was obtained by chart review, consisting of both patient and treatment factors, such as age, preoperative SHIM score^28^, ASA physical status^29^, NS extent, etc. (Table 1). Follow-up data at 12 months were obtained by chart review or telephone by an independent research coordinator utilizing patient-reported outcomes.

### Video annotation

Bilateral NS video footage was manually reviewed. A total of 7 annotators (RM, IR, GD, AD, SC, MO, SR) received standardized training and then independently labeled gesture sequences of three training videos (365 gestures in total). The gesture classification agreement rate among seven annotators was evaluated by calculating the proportion of gesture labels agreed upon among all 7 annotators in the total number of gestures. A high inter-rater agreement rate was achieved (328/365, 89.9%), and then 80 formal NS videos were split and annotated amongst annotators.

Every discrete surgical movement in the video was labeled as a certain gesture according to our classification system, which includes nine active dissection gestures and 4 supporting gestures (i.e., gestures intended to facilitate dissection gestures, e.g., retraction) (Fig. 1)^5^. When more than one instrument moved simultaneously, the movement of the dominant-hand instrument of the console surgeon was annotated as the primary gesture.

### Traditional statistical analysis

Mann–Whitney U- and chi-square tests were used to compare continuous and categorical patient demographic data, respectively. A multi-level mixed-effects model was used to evaluate the relationship between 1-year EF recovery status (independent variable) and the proportion of each type of gesture within a case (dependent variable), while accounting for data clustering given that multiple cases were done by the same surgeon. The relationship between surgeon experience (independent variable) and the proportion of each type of gesture within a case (dependent variable) was also evaluated by the multi-level mixed-effects model to identify dissection technique differences. Statistical analysis was conducted using IBM^®^ SPSS v24, with *p* < 0.05 (two-sided) considered as statistically significant.

### Machine learning model construction

Gesture sequences (i.e., all gestures used in NS in the order of time) and clinical features (i.e., all variables shown in Table 1) were both used to construct prediction models for 1-year EF recovery. To confirm the reproducibility of results, two ML teams independently constructed prediction models using ML algorithms and tested model performance.

ML Team 1 (JX, LT, LY) trained a multi-modal prediction model, consisting of two subnetworks used to handle the entire gesture sequences (a transformer-based network, i.e., IMV-LSTM^30^) and clinical features (an FT Transformer, i.e., tabular network for the clinical features^31^). The networks were chosen due to their attention mechanisms, which are modules that learn to calculate the weighted sum of all encoded gesture representation vectors, allowing the model to flexibly capture long-term dependencies and focus its attention on the most relevant parts of the entire dissection sequence. In the first phase of training, both subnetworks were trained until convergence with stochastic gradient descent. In the second phase, the representations extracted from each network were concatenated and fed into a fully connected layer to yield a single EF recovery prediction. The model was then evaluated by a Monte-Carlo method with a total of 100 iterations. In each iteration, we randomly selected 70 cases as the training data and the remaining 10 cases as the hold-out set to independently test the model performance. We report the area-under-the-ROC-curve (AUC) and 95% confidence interval (CI) of the test set across the 100 iterations. To illustrate important sequences for EF prediction, Team 1 extracted attention scores for each gesture within a sequence and occlusion techniques were used to extract directionality as an indicator for gesture sequences that correlated positively or negatively with EF recovery.

ML Team 2 (DK, AA) constructed a logistic regression prediction model for 1-year EF recovery. This model was chosen due to its simplicity and to avoid memorizing the data (i.e., overfitting). When considering the clinical features alone, the logistic regression model directly mapped such features to 1-year EF recovery outcome. When considering the gesture sequence alone, Team 2 employed a weakly-supervised approach. This involved splitting the entire gesture sequence into nonoverlapping, equally-sized segments comprising 20 gestures (the number of gestures per segment was empirically determined on a held-out set). During the training phase of the model, each segment was mapped onto the corresponding case-specific 1-year EF recovery outcome. For example, if a case has 440 gestures, this would result in 440/20 = 22 subsequences of gestures. Each subsequence can equivalently be thought of as a distinct sample in a database. With these subsequences belonging to the same surgical case and a surgical case being associated with a single target (i.e., EF recovery), we used the case’s target for all such subsequences. This would result in 22 input-output pairs consisting of input gesture subsequences and output EF recovery values. We repeated this strategy for all surgical cases in order to generate the complete database on which the logistic regression would be trained on. Such a setup is referred to as ‘weakly-supervised learning’ and is often adopted in order to expand the size of the dataset on which a model is trained. Note that for gesture sequences whose length was not divisible by 20, the tail-end of the sequence of gestures was dropped and thus not presented to the model. This is because a logistic regression model expects inputs of a consistent dimension. The model was trained on the aforementioned database of gesture subsequences and EF recovery values. Given a gesture subsequence (comprising 20 gestures), the model returned a single prediction reflecting whether or not the patient will recover EF at 1 year. These 20 gestures do not capture the entire action performed by the surgeon during the NS step. To capture all such action during inference, as is common with weakly-supervised learning, we aggregate all model predictions for subsequences that belong to the same surgical case. We implemented a majority rule where the most likely prediction across all case-specific samples was considered as the final prediction for that particular surgical case. For example, if 15/22 samples are associated with an EF recovery prediction, then the model predicts this case will recover EF at 1 year. When considering both the dissection gesture sequence and the clinical features, this team continued to employ the aforementioned weakly-supervised approach. Team 2 implemented the same evaluation setup as Team 1 and reported AUC with 95% CI across the 100 iterations.

### Reporting summary

Further information on research design is available in the Nature Research Reporting Summary linked to this article.

## Supplementary information

Supplementary materials

Reporting Summary

## Data Availability

The datasets generated during and/or analyzed during the current study are available from the corresponding author on reasonable request.

## Code availability

The code of this article can be found by: https://github.com/crseusc/NS-Gestures-and-EF-outcomes.
