# Supplementary material for: Surgical gestures as a method to quantify surgical performance and predict patient outcomes
Source: NPJ Digit Med. 2022 Dec 22;5:187. doi: 10.1038/s41746-022-00738-y (PMC9780308; doi:10.1038/s41746-022-00738-y)
Supplement: Supplementary file 1 — Supplementary materials [file 41746_2022_738_MOESM1_ESM.pdf]

Supplementary Table 1. Demographics of surgeons included in this study

**Features**<br>Median (range)/Number	**Super-experts**<br>(N=6)	**Experts**<br>(N=15)
Prior robotic surgical caseload	3000 (2000-5800)	275 (100-750)
Attending/Fellow	6/0	11/4
Cases contributed to the study	53	27

Supplementary Table 2. Separating all cases into four quartiles based on the amount of gestures per case, and comparing 1-year EF recovery rate among groups (p=0.66, Chi-square test).

	**No. of patients who recovered EF at 1 year**	**No. of patients who did not recover EF at 1 year**
Quartile 1 *(least gestures)*	11 (55%)	9 (45%)
Quartile 2	11 (52%)	10 (48%)
Quartile 3	13 (68%)	6 (32%)
Quartile 4 *(most gestures)*	10 (50%)	10 (50%)

Supplementary Table 3. Comparison of clinical features of patients between experts and super-experts

Features	Experts Median (IQR) / Count (%) (N = 27)	Super-experts Median (IQR) / Count (%) (N = 53)	P value
**Patient factors**			
Age, year	64 (59-68)	63 (59-67)	1.00
BMI, kg/m^2	27.6 (25.7-30.8)	28.1 (25.8-29.8)	0.59
Preop SHIM score	24 (19-25)	24 (22-25)	0.43
PSA, ng/mL	8.4 (6.5-11.7)	6.2 (5.2-9.8)	0.31
ASA			0.84
I	4 (14.8%)	7 (13.2%)	
≥II	23 (85.2%)	46 (86.8%)	
Pre-op Gleason score			0.06
6 (ISUP 1)	11 (40.7%)	9 (17.0%)	
7 (ISUP 2/3)	11 (40.7%)	33 (62.3%)	
≥8 (ISUP 4/5)	5 (18.5%)	11 (20.8%)	
Post-op Gleason score			0.63
6 (ISUP 1)	4 (14.8%)	6 (11.3%)	
7 (ISUP 2/3)	20 (74.1%)	37 (69.8%)	
≥8 (ISUP 4/5)	3 (11.1%)	10 (18.9%)	
Pathological tumor stage			0.24
pT2	16 (59.3%)	24 (45.3%)	
≥pT3	11 (40.7%)	29 (54.7%)	
Prostate volume, g	50 (33-67)	39 (34-53)	0.49
**Treatment factors**			
Nerve Sparing Extent			0.76
Partial	8 (29.6%)	14 (26.4%)	
Full	19 (70.4%)	39 (73.6%)	
**Outcomes**			
1-yr EF Recovery			0.82
Yes	16 (40.7%)	23 (43.4%)	
No	11 (59.3%)	30 (56.6%)	

Continuous variables were compared by Mann-Whitney U test and reported as median (IQR). Categorical variables were compared by Chi-square test or Fisher exact test as indicated. ASA, American Society of Anesthesiology physical status classification system; BMI, Body Mass Index; IQR, Interquartile Range; SHIM, Sexual Health Inventory for Men; ISUP, International Society of Urological Pathology; PSA, Prostate Specific Antigen.

TRIPOD Checklist: Prediction Model Development and Validation

Section/Topic	Item		Checklist Item	Page
**Title and abstract**				
Title	1	D;V	Identify the study as developing and/or validating a multivariable prediction model, the target population, and the outcome to be predicted.	1
Abstract	2	D;V	Provide a summary of objectives, study design, setting, participants, sample size, predictors, outcome, statistical analysis, results, and conclusions.	4
**Introduction**				
Background and objectives	3a	D;V	Explain the medical context (including whether diagnostic or prognostic) and rationale for developing or validating the multivariable prediction model, including references to existing models.	5
	3b	D;V	Specify the objectives, including whether the study describes the development or validation of the model or both.	6
**Method**				
Source of data	4a	D;V	Describe the study design or source of data (e.g., randomized trial, cohort, or registry data), separately for the development and validation data sets, if applicable.	14
	4b	D;V	Specify the key study dates, including start of accrual; end of accrual; and, if applicable, end of follow-up.	14
Participants	5a	D;V	Specify key elements of the study setting (e.g., primary care, secondary care, general population) including number and location of centres.	14
	5b	D;V	Describe eligibility criteria for participants.	14
	5c	D;V	Give details of treatments received, if relevant.	14
Outcome	6a	D;V	Clearly define the outcome that is predicted by the prediction model, including how and when assessed.	15
	6b	D;V	Report any actions to blind assessment of the outcome to be predicted.	15
Predictors	7a	D;V	Clearly define all predictors used in developing or validating the multivariable prediction model, including how and when they were measured.	16
	7b	D;V	Report any actions to blind assessment of predictors for the outcome and other predictors.	17
Sample size	8	D;V	Explain how the study size was arrived at.	
Missing data	9	D;V	Describe how missing data were handled (e.g., complete-case analysis, single imputation, multiple imputation) with details of any imputation method.	NA
Statistical analysis methods	10a	D	Describe how predictors were handled in the analyses.	16-18
	10b	D	Specify type of model, all model-building procedures (including any predictor selection), and method for internal validation.	16-18
	10c	V	For validation, describe how the predictions were calculated.	NA
	10d	D;V	Specify all measures used to assess model performance and, if relevant, to compare multiple models.	16-18
	10e	V	Describe any model updating (e.g., recalibration) arising from the validation, if done.	NA
Risk groups	11	D;V	Provide details on how risk groups were created, if done.	NA
Development vs. validation	12	V	For validation, identify any differences from the development data in setting, eligibility criteria, outcome, and predictors.	NA
**Results**				
Participants	13a	D;V	Describe the flow of participants through the study, including the number of participants with and without the outcome and, if applicable, a summary of the follow-up time. A diagram may be helpful.	6
	13b	D;V	Describe the characteristics of the participants (basic demographics, clinical features, available predictors), including the number of participants with missing data for predictors and outcome.	6
	13c	V	For validation, show a comparison with the development data of the distribution of important variables (demographics, predictors and outcome).	7
Model development	14a	D	Specify the number of participants and outcome events in each analysis.	7
	14b	D	If done, report the unadjusted association between each candidate predictor and outcome.	NA
Model specification	15a	D	Present the full prediction model to allow predictions for individuals (i.e., all regression coefficients, and model intercept or baseline survival at a given time point).	NA
	15b	D	Explain how to the use the prediction model.	8
Model performance	16	D;V	Report performance measures (with CIs) for the prediction model.	8
Model-updating	17	V	If done, report the results from any model updating (i.e., model specification, model performance).	NA
**Discussion**				
Limitations	18	D;V	Discuss any limitations of the study (such as nonrepresentative sample, few events per predictor, missing data).	13
Interpretation	19a	V	For validation, discuss the results with reference to performance in the development data, and any other validation data.	NA
	19b	D;V	Give an overall interpretation of the results, considering objectives, limitations, results from similar studies, and other relevant evidence.	11-12
Implications	20	D;V	Discuss the potential clinical use of the model and implications for future research.	13
**Other information**				
Supplementary information	21	D;V	Provide information about the availability of supplementary resources, such as study protocol, Web calculator, and data sets.	19
Funding	22	D;V	Give the source of funding and the role of the funders for the present study.	21

*Items relevant only to the development of a prediction model are denoted by D, items relating solely to a validation of a prediction model are denoted by V, and items relating to both are denoted D;V. We recommend using the TRIPOD Checklist in conjunction with the TRIPOD Explanation and Elaboration document.
