# Supplementary material for: Surgical gestures as a method to quantify surgical performance and predict patient outcomes
Source: NPJ Digit Med. 2022 Dec 22;5:187. doi: 10.1038/s41746-022-00738-y (PMC9780308; doi:10.1038/s41746-022-00738-y)
Supplement: Supplementary file 2 — Reporting Summary [file 41746_2022_738_MOESM2_ESM.pdf]

nature portfolio

Corresponding author(s): Andrew J. Hung

Last updated by author(s): Oct 22, 2022

Reporting Summary

Nature Portfolio wishes to improve the reproducibility of the work that we publish. This form provides structure for consistency and transparency in reporting. For further information on Nature Portfolio policies, see our Editorial Policies and the Editorial Policy Checklist.

Statistics

For all statistical analyses, confirm that the following items are present in the figure legend, table legend, main text, or Methods section.

n/a	Confirmed	
☐	☒	The exact sample size (n) for each experimental group/condition, given as a discrete number and unit of measurement
☐	☒	A statement on whether measurements were taken from distinct samples or whether the same sample was measured repeatedly
☐	☒	The statistical test(s) used AND whether they are one- or two-sided<br>*Only common tests should be described solely by name; describe more complex techniques in the Methods section.*
☐	☒	A description of all covariates tested
☐	☒	A description of any assumptions or corrections, such as tests of normality and adjustment for multiple comparisons
☐	☒	A full description of the statistical parameters including central tendency (e.g. means) or other basic estimates (e.g. regression coefficient) AND variation (e.g. standard deviation) or associated estimates of uncertainty (e.g. confidence intervals)
☐	☒	For null hypothesis testing, the test statistic (e.g. F, t, r) with confidence intervals, effect sizes, degrees of freedom and P value noted<br>*Give P values as exact values whenever suitable.*
☒	☐	For Bayesian analysis, information on the choice of priors and Markov chain Monte Carlo settings
☐	☒	For hierarchical and complex designs, identification of the appropriate level for tests and full reporting of outcomes
☒	☐	Estimates of effect sizes (e.g. Cohen's d, Pearson's r), indicating how they were calculated

Our web collection on statistics for biologists contains articles on many of the points above.

Software and code

Policy information about availability of computer code

Data collection	Excel V16
Data analysis	SPSS V24 was used for traditional stats and Python was used for machine learning models building. Codes were uploaded to https://github.com/crseusc/NS-Gestures-and-EF-outcomes.

For manuscripts utilizing custom algorithms or software that are central to the research but not yet described in published literature, software must be made available to editors and reviewers. We strongly encourage code deposition in a community repository (e.g. GitHub). See the Nature Portfolio guidelines for submitting code & software for further information.

Data

Policy information about availability of data

All manuscripts must include a data availability statement. This statement should provide the following information, where applicable:

- Accession codes, unique identifiers, or web links for publicly available datasets
- A description of any restrictions on data availability
- For clinical datasets or third party data, please ensure that the statement adheres to our policy

The datasets generated during and/or analysed during the current study are available from the corresponding author on reasonable request. Codes can be found by https://github.com/crseusc/NS-Gestures-and-EF-outcomes.

Human research participants

Policy information about studies involving human research participants and Sex and Gender in Research.

Reporting on sex and gender	Prostate cancer can only happen in men, thus we did not perform analysis based on sex.
Population characteristics	All patients were men, age 63 (IQR 59-67), with preoperative potency, diagnosed as prostate cancer and underwent robot-assisted radical prostatectomy (RARP).
Recruitment	619 consecutive RARP cases from July 2016 to November 2018 were candidates for this study, and eventually 80 cases from 21 surgeons from 2 international surgical centers fulfilled our inclusion/exclusion criteria. Most patients were excluded because they did not have baseline erectile function to be preserved during surgery.
Ethics oversight	University of Southern California and St. Antonius-Hospital Review Board

Note that full information on the approval of the study protocol must also be provided in the manuscript.

Field-specific reporting

Please select the one below that is the best fit for your research. If you are not sure, read the appropriate sections before making your selection.

☒ Life sciences ☐ Behavioural & social sciences ☐ Ecological, evolutionary & environmental sciences

For a reference copy of the document with all sections, see nature.com/documents/nr-reporting-summary-flat.pdf

Life sciences study design

All studies must disclose on these points even when the disclosure is negative.

Sample size	No sample size calculation was performed and sample size was determined experientially.
Data exclusions	619 consecutive RARP cases from July 2016 to November 2018 were candidates for this study, and eventually 80 cases from 21 surgeons from 2 international surgical centers fulfilled our inclusion/exclusion criteria. Most patients (N=331) were excluded because they did not have baseline erectile function to be preserved during surgery. Other reasons are declined to participate (N=134), loss of follow up (N=37), incomplete surgical videos (n=34), and non-nerve sparing cases (N=3).
Replication	Results here are reproducible because we have two machine-learning teams independently confirmed our results.
Randomization	This is a prospective observational study so no randomization was performed.
Blinding	Investigators were blinded to surgeon identity when they were annotating surgical videos, which increased the reliability of gesture annotation and keep the annotation unbiased.

Reporting for specific materials, systems and methods

We require information from authors about some types of materials, experimental systems and methods used in many studies. Here, indicate whether each material, system or method listed is relevant to your study. If you are not sure if a list item applies to your research, read the appropriate section before selecting a response.

Materials & experimental systems

n/a	Involved in the study	
☒	☐	Antibodies
☒	☐	Eukaryotic cell lines
☒	☐	Palaeontology and archaeology
☒	☐	Animals and other organisms
☐	☒	Clinical data
☒	☐	Dual use research of concern

Methods

n/a	Involved in the study	
☒	☐	ChIP-seq
☒	☐	Flow cytometry
☒	☐	MRI-based neuroimaging

Clinical data

Policy information about clinical studies

All manuscripts should comply with the ICMJE guidelines for publication of clinical research and a completed CONSORT checklist must be included with all submissions.

Clinical trial registration	Clinical trial registration can be found here - https://reporter.nih.gov/search/ws0dai_A7EeitJW3MTUOtg/project-details/10497797#details
Study protocol	Protocol can be found here - https://reporter.nih.gov/search/ws0dai_A7EeitJW3MTUOtg/project-details/10497797#details
Data collection	Clinical data was obtained by chart review, consisting of both patient and treatment factors, such as age, preoperative SHIM score , ASA physical status, NS extent, etc. Follow-up data at 12 months were obtained by chart review or telephone by an independent research coordinator utilizing patient-reported outcomes.
Outcomes	The primary outcome was 1-year EF recovery after RARP. 1-year EF recovery were both defined as achieving erections firm enough for sexual intercourse in >50% of attempts (score of ≥4 on the 2nd questions of the SHIM) with or without phosphodiesterase type 5 inhibitors.
